# Fluorogenic properties of 4-dimethylaminocinnamaldehyde (DMACA) enable high resolution imaging of cell-wall-bound proanthocyanidins in plant root tissues

**DOI:** 10.3389/fpls.2022.1060804

**Published:** 2023-01-16

**Authors:** Jamil Chowdhury, Jannatul Ferdous, Jenna Lihavainen, Benedicte Riber Albrectsen, Judith Lundberg-Felten

**Affiliations:** ^1^ Umeå Plant Science Center, Department of Plant Physiology, Umeå University, Umeå, Sweden; ^2^ Umeå Plant Science Center, Department of Forest Genetics and Plant Physiology, Swedish University of Agricultural Sciences, Umeå, Sweden

**Keywords:** cell-wall-bound proanthocyanidin, 4-dimethylaminocinnamaldehyde (DMACA), fluorescence spectra determination, flavan-3-ol, localization, plant fluorescent dye characterization

## Abstract

Proanthocyanidins (PAs) are polymeric phenolic compounds found in plants and used in many industrial applications. Despite strong evidence of herbivore and pathogen resistance-related properties of PAs, their *in planta* function is not fully understood. Determining the location and dynamics of PAs in plant tissues and cellular compartments is crucial to understand their mode of action. Such an approach requires microscopic localization with fluorescent dyes that specifically bind to PAs. Such dyes have hitherto been lacking. Here, we show that 4-dimethylaminocinnamaldehyde (DMACA) can be used as a PA-specific fluorescent dye that allows localization of PAs at high resolution in cell walls and inside cells using confocal microscopy, revealing features of previously unreported wall-bound PAs. We demonstrate several novel usages of DMACA as a fluorophore by taking advantage of its double staining compatibility with other fluorescent dyes. We illustrate the use of the dye alone and its co-localization with cell wall polymers in different *Populus* root tissues. The easy-to-use fluorescent staining method, together with its high photostability and compatibility with other fluorogenic dyes, makes DMACA a valuable tool for uncovering the biological function of PAs at a cellular level in plant tissues. DMACA can also be used in other plant tissues than roots, however care needs to be taken when tissues contain compounds that autofluoresce in the red spectral region which can be confounded with the PA-specific DMACA signal.

## Introduction

Proanthocyanidins (PAs or condensed tannins) are ubiquitous plant-defense-related secondary metabolites of phenolic nature commonly found in woody plants and forest trees (Ferreira and Li, 2000). PAs are end-products of the flavonoid biosynthetic pathway. They are composed of oligomeric and/or polymeric units of flavan-3-ol monomers, with catechin and epicatechin being the basic building blocks (Rue et al., 2017; Rauf et al., 2019). Although PA structures vary greatly between plant species, they are generally classified as A-type or B-type PAs based on their detailed chemical structure (Ferreira and Slade, 2002). B-type PAs can be converted to A-type PAs by oxidation. The central vacuole is thought to be the primary location of deposition after biosynthesis (Dixon and Sarnala, 2020). Increasing evidence suggests that a considerable number of PAs are also potentially cross-linked with cell wall polymers (Dixon and Sarnala, 2020). The biosynthesis of PAs is relatively well understood (Zhao and Dixon, 2009; Zhao, 2015; James et al., 2017). The transport of PA precursors and the cellular localization of polymerization reactions may, however, depend on the plant species studied and needs further investigation (Dixon and Sarnala, 2020). The authors of the latter review have emphasized poplar as an ideal plant species for this, alongside with tea and grapevine. Evidence is accumulating that PAs play an important *in planta* role in plant protection, e.g., acting against leaf-eating herbivores, fungal pathogens and abiotic stress (UV protection) (Constabel et al., 2014; Dixon and Sarnala, 2020; Ullah et al., 2019). However, it remains unclear how PAs are incorporated into plant cell walls and what their function is under various developmental and stress conditions. Development of suitable analytical methods is key to solving this issue.

Several wet lab-based methods are well established for PA analysis. These include, but are not limited to, acid butanol assays, chromatographic methods, LC-MS, LC-MS/MS, MALDI-TOF-MS and NMR (Hümmer and Schreier, 2008; Porter et al., 1985). While many of these methods can achieve high sensitivity and reliability when analyzing PAs, they are unsuitable for determining *in situ* PA levels at the (sub-)cellular level due to the requirement for tissue maceration during sample processing. Understanding the function and dynamics of PAs in cell walls *in situ* requires the use of high-resolution microscopy coupled with PA-specific fluorescent dyes. Chromophore dyes vanillin and 4-dimethylaminocinnamaldehyde (DMACA) have specificity for PAs and have been used in light microscopy. PAs produce pink precipitates in reaction with vanillin (Gardner, 1975; Ferreira et al., 2017) and dark blue precipitates in reaction with DMACA (Abeynayake et al., 2011; Ferreira et al., 2017). DMACA-based assays have been shown to have up to 5-fold higher sensitivity to PAs compared to the vanillin assay (Hümmer and Schreier, 2008). The sensitivity of DMACA to flavan-3-ols is so high that all other potentially interfering substances in plant cells (e.g., caffeine, anthocyanins, chlorogenic acid, citric acid, gallic acid, and rutin) would require approximately 100 to 1000 fold their concentration to produce similar absorbance (Treutter, 1989; Wallace and Giusti, 2010). Although both dyes allow convenient visualization of PAs in vacuoles in light microscopy, the images are neither quantitative nor sufficiently resolved to visualize cell-wall-bound PAs. Interestingly, both vanillin and DMACA have fluorogenic properties that have been extensively exploited in material sciences unrelated to plant biology (Kaito et al., 1979; Katayama et al., 1987; Dryjanski et al., 1999; Singh and Mitra, 2008; Fritz et al., 2015; Poojary et al., 2019). DMACA fluorescence relies on an intramolecular charge transfer (ICT) mechanism, which results in substrate-specific excitation and emission spectra (Singh and Mitra, 2008). Owing to its fluorogenic properties and high sensitivity to PAs, we explored the PA-specific fluorogenic properties of DMACA *in situ* for possible use in high resolution microscopy in plant sciences. We tested its compatibility with other fluorescent dyes for the co-localization of PAs with other cell wall polymers. Using spectral scanning, we confirmed the spectral properties of PA-specific DMACA fluorescence under both *in vitro* and *in situ* conditions.

## Materials and methods

### Biological materials

All root samples used in this study were collected from *in vitro* grown hybrid aspen plants (*Populus tremula* x *Populus tremuloides*, T89) prepared and grown as described in Chowdhury et al. (2022). Ectomycorrhizal roots were developed by *in vitro* interactions of T89 and *Laccaria bicolor* (isolate S238N) fungus using a sandwich culture system (Felten et al., 2009; Chowdhury et al., 2022). Leaf samples were collected from twigs at the lower end of field-grown Swedish Aspen Collection (SwAsp) lines in June. Leaves in the second and third positions from the top of the twigs were used for the study. The line ID number corresponds to the SwAsp ID as described in Bandau et al. (2015). The six SwAsp lines selected for this study had previously been reported by Bandau et al. (2015) to possess either low (SwAsp ID 23, 60, and 115) or high (SwAsp ID 5, 65, and 117) levels of foliar PAs.

### Reagents

DMACA and all PA compounds (**Supplementary Figure 1**) used in this study were bought from Sigma-Aldrich, Sweden AB, Stockholm: 4-dimethylaminocinnamaldehyde (Cat. # D4506), Procyanidin A2 (Cat. # 28660), procyanidin B2 (Cat. # 42157), (-)-epigallocatechin (Cat. # 08108) and (+)-catechin (Cat. # 43412). All PA compounds were solubilized in DMSO.

### PA extraction, isolation and characterization

PAs were isolated from roots of one-year-old hybrid aspen plants (*Populus tremula x Populus tremuloides*, T89) grown in a greenhouse. Isolation and purification steps were performed according to methods described in Shay et al. (2017) and Hagerman (2002) with some modifications. Roots were lyophilized, ground to fine powder with a mortar and a pestle and 6 g of root powder were used for PA extraction. Crude PA extraction was performed with 70% aqueous acetone (containing 0.01% ascorbic acid). Samples were vortexed, sonicated in a water bath (30 min, 15°C) and centrifuged (5500 ×g, 15 min, 4°C). Crude PA extract was mixed with an equal volume of hexane in a separation funnel to remove lipophilic compounds (waxes and fatty acids etc.). The aqueous phase was collected and dried under a stream of air, resuspended in 95% ethanol and applied onto a Sephadex LH-20 column for the separation of PAs from other phenolics and carbohydrates. The column was prepared as instructed by the manufacturer (Sigma-Aldrich, Sweden AB, Stockholm) by mixing Sephadex LH-20 with ethanol (EtOH) and settled for three hours. Settled media was mixed with EtOH (75:25) and the slurry was poured into the glass column with a sintered glass filter at the bottom. After equilibration of the sephadex column with EtOH, the PA extract was poured into the column. The column was then washed with EtOH and the eluted fractions were collected and monitored with Prussian blue assay and reducing carbohydrate assay to confirm the removal of phenolics and carbohydrates. When no phenolics and carbohydrates were any longer detected in the eluate, the Sephadex-bound-PAs were eluted with 70% acetone. The fractions were collected and monitored by acid-butanol assay (Porter et al., 1985) using Procyanidin B2 as a standard to confirm the presence of PAs. The fractions enriched with PAs were combined and dried under vacuum and weighed (the final PA powder weight was 54 mg). For compositional analysis of the isolated PA sample the PA fraction isolated from hybrid aspen roots was dissolved in 50% MeOH containing internal standards (**Supplementary Table S1**) and the composition of monomers and oligomers in the PA sample (injected 2 µL) were analysed with UPLC-MS-ESI-QTOF in positive and negative ionization modes. The compounds were separated in a Waters Acquity HSS T3 C18 column (2.1 × 50 mm, 1.8 µm) equipped with a VanGuard pre-column (2.1 5 mm, 1.8 µm, Waters, Milford, MA, USA) at an oven temperature of 40°C. An Agilent 1290 Infinity UPLC system was equipped with an Agilent 6546 Q-TOF mass spectrometer (Agilent Technologies). The elution solvents were water with 0.1% of formic acid (A) and acetonitrile with 0.1% of formic acid (B), a flow rate was 0.5 ml/min and the gradient was following: from 10% B to 99% B in 7 min and at 99% B for 2 min. The mass range was m/z 70-1700. Data processing was performed with Agilent MassHunter Profinder software (version 10.0, Agilent Technologies Inc., Santa Clara, CA, USA).

### *In vitro* characterization of DMACA spectra in the presence of PAs

#### Pas photospectrometry

The DMACA reagent was prepared by solubilizing 0.05% (w/v) DMACA in absolute ethanol containing 0.8% (w/v) hydrochloric acid. Individual PA compounds were added to the DMACA reagent at a final concentration of 0.5 mg/mL, and the solution was incubated for 30 minutes at room temperature. The absorbance spectra of the solution were recorded in a 96-well microplate reader spectrophotometer (Epoch Biotek) with 100 µl per well (optical pathlength is approximately 0.31 cm) using the pathlength correction function to determine the exact volume in each well. The measured absorbance values were then normalized to 1 cm pathlength. Immediately after, the fluorescence excitation and emission spectra of the samples were measured using a fluorimeter (BioTek™ Synergy™ H4 hybrid microplate reader). Excitation scans were performed from 550 to 750 nm at 10 nm increments with the emission wavelength set to 770 nm. Emission scans were performed from 580 to 800 at 10 nm increments with the excitation wavelength set to 550 nm. The experiment was repeated twice with four technical replicates, which yielded very similar results.

#### Fluorescent spot test

PAs and cell wall polysaccharides were mixed with the DMACA reagent described above. For the tested PAs the highest concentration (1 mg/ml) corresponds to 3.4 mM for (+)-Catechin, 1.7 mM for Procyanidin B2, 3.3 mM for (i)-Epigallocathechin and 1.7 mM for Procyanidin A2. After 30 minutes of incubation at room temperature, 2 µL of the mixed reagents were loaded onto a PVDF membrane (Cat# IPVH00010, Merkmillipore) and images were recorded immediately with a fluorescent western blot imaging system (Azure 600c, Azure Biosystems) using three RGB fluorescence channels: Cy2 (*λ*_ex_/*λ*_em_= 492 nm/510 nm), Cy3 (*λ*_ex_/*λ*_em_= 550 nm/570 nm) and Cy5(*λ*_ex_/*λ*_em_= 650 nm/670 nm). The experiment was repeated twice, providing similar results.

### *In situ* DMACA fluorescence characterization

#### DMACA staining

For root samples, freshly harvested poplar root tips were dehydrated in an ethanol series of increasing concentration (30%, 50%, 70% and 100%, 10 minutes in each dilution). Samples were incubated for 15 minutes in DMACA reagent (0.02% w/v DMACA in absolute ethanol containing 0.8% w/v hydrochloric acid) as described in previous studies (Li et al., 1996; Abeynayake et al., 2011). The appearance of a blue/purple precipitate indicated that PAs were present in the material. Following DMACA staining, samples were rehydrated in an ethanol series of decreasing concentration (100%, 70%, 50%, 30% and 0%, 10 minutes in each dilution) and kept in PBS solution before being embedded in 4% agarose in PBS. Agarose embedded samples protected from light could be kept in the fridge for a few days before sectioning. Roots and leaves were cross sectioned at 70 µm thickness using a vibratome (Leica VT1000S). Sections were then transferred onto microscopy glass slides, mounted in 50% glycerol and protected from light before microscopic observation. The experiment was conducted using three to four roots collected from three to four plants. Leaves were stained with DMACA reagent following the same procedure as for root staining; additionally, they were left to dehydrate in the ethanol series for 20 minutes in each dilution to remove chlorophyll.

#### Spectral analysis

Simultaneous excitation and emission (lambda-lambda scan) was performed on DMACA-stained poplar root cross-sections using a Leica Stellaris confocal laser-scanning microscope (Leica Microsystems, Mannheim, Germany). A tunable white light laser, calibrated to have a steady output power over the available spectrum, was used as the excitation source. Excitation scans were recorded every 10 nm between 489 nm and 659 nm.

#### Photobleaching assay

DMACA and Calcofluor White photobleaching properties were determined by continuously exposing dye-stained root cross-sections to 633 nm laser light and 405 nm laser light, respectively, and micrographs were taken at the times indicated in the Results section. Fluorescence intensity was measured from at least ten individual areas at each timepoint. The signal intensity was normalized to the signal at time zero (considered 1), and the photobleaching curve was constructed by averaging the relative intensity at the respective times.

#### Counter staining and high-resolution microscopy

Three to four lateral roots collected from three to four *in vitro* grown *Populus tremula x Populus tremuloides* plants were stained with 0.02% DMACA and sectioned at 70 µm deep, as described earlier. For counterstaining with Calcofluor White, DMACA-stained root sections were incubated with 100 µL Calcofluor White M2R (Cat. # 910090, Sigma-Aldrich) stock solution for 5 min at room temperature, briefly washed with water and mounted on a glass slide. For counterstaining with Auramine O, DMACA-stained root sections were incubated with 0.1% Auramine O (Cat. # A9655, Sigma) solubilized in 50 mM Tris-HCl, pH 7.3, for 15-20 minutes at room temperature, protecting from light. The sections were then briefly washed with 50 mM Tris-HCl and mounted on glass slides. Images were acquired with a Zeiss LSM 880 microscope operated in Airyscan mode (Huff, 2015) using 633 nm (DMACA fluorescence), 488 nm (Auramine O fluorescence) and 405 nm (Calcofluor white fluorescence) laser lines, MBS 458/514/561/633 nm beam splitters and a C-Apochromat 40x/1.2 W Korr FCS M27 objective.

## Results

### DMACA has PA-specific fluorescence excitation and emission

**Figure 1:**
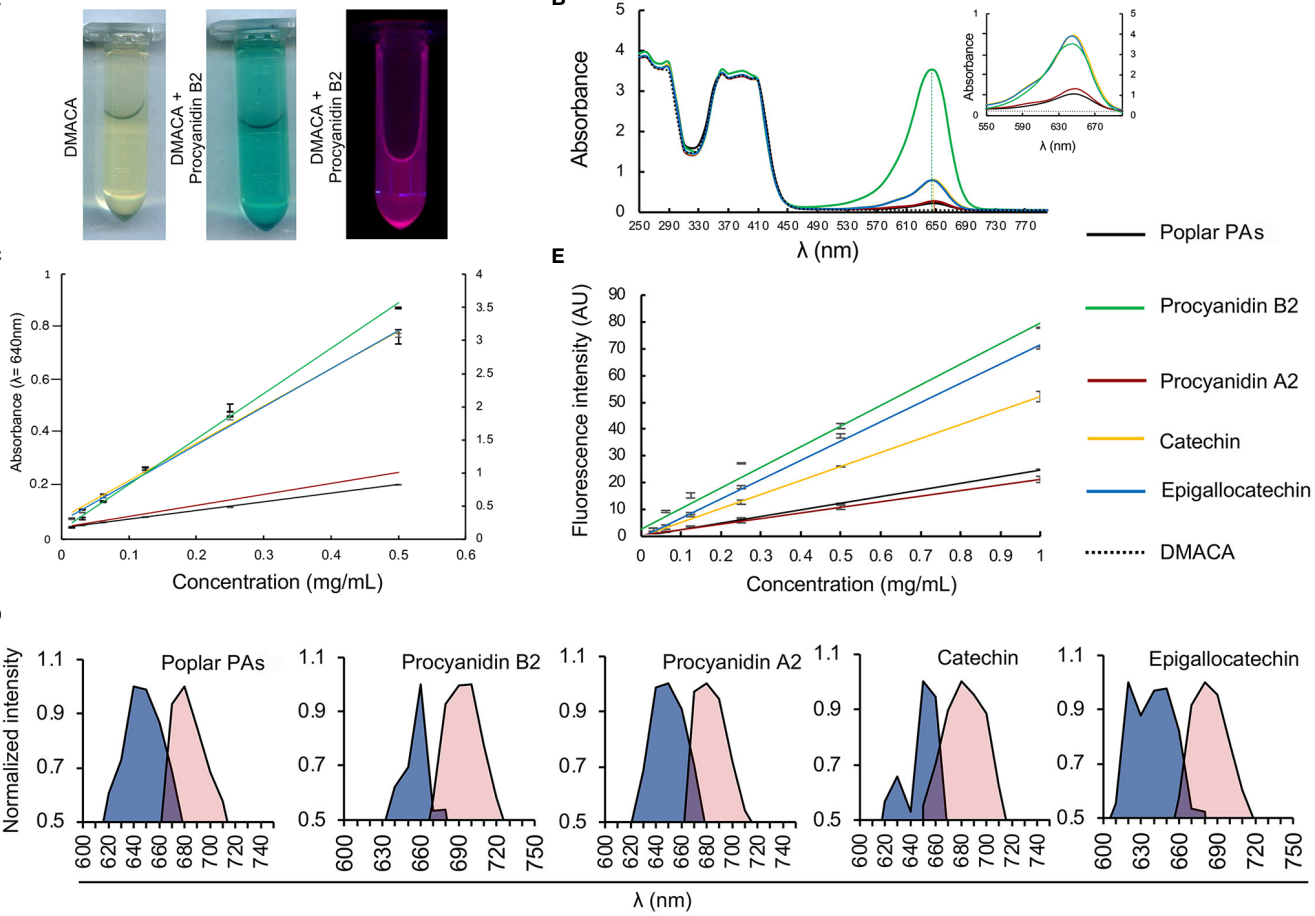
DMACA fluorogenic properties. **(A)** DMACA yields blue precipitates in the presence of PAs (in this example with procyanidin B2, tube in the centre), which emits fluorescence in a fluorometer (purple, tube furthest to the right). **(B)** In a spectrophotometer, DMACA alone has absorbance peaks at 250 ± 20 nm and 390 ± 30 nm. In the presence of PA species, (procyanidin A2, B2, catechin, epigallocatechin, and poplar PA-extract), the DMACA absorbance spectrum exhibits a characteristic additional peak at 650 ± 40 nm. The position of peak maxima are indicated by vertical dashed lines. In the inset in **(B)** as well as in **(C)** the primary Y axis to the left of each graph applies to Procyanidin A2, Catechin, Epigallocatechin, and the poplar PA-extract and the secondary Y axis on the right applies to Procyanidin B2 for better visualization without overlapping curves. **(C)** PA quantification using a standard curve at 640nm with data from **(B)**. **(D)** In the fluorimeter, DMACA-bound excitation spectra (blue area) and emission spectra (red area) differ among PA species. **(E)** DMACA fluorescence data (*λ*_ex_ = 650 nm, *λ*_em_ = 690 nm, cutoff filter 665 nm) can be used for quantification of PA species.

We investigated the PA-specific fluorogenic properties of DMACA in an ethanol-based acidic reagent in the presence of four commercial PAs commonly present in plant cells, namely procyanidin B2, procyanidin A2, catechin and epigallocatechin, along with isolated PAs from poplar roots. The PA fraction from hybrid aspen roots consisted of catechin and gallocatechin monomers and a complex mixture of their oligomers. Procyanidins (comprising of catechin subunits) were more abundant than prodelphinidins (gallocatechin subunits). The largest PA polymers detected with UPLC-MS were pentamers and the major constituents were procyanidin trimers based on the peak area (**Supplementary Tables 1, 2**; **Supplementary Figure 2**). DMACA exhibited a characteristic spectrophotometric absorbance peak within the range 590 to 690 nm only in the presence of the tested compounds, with peaks (*λ*_max_) at 640 nm to 650 nm. PAs alone in EtOH-HCl do not show any absorbance in this region (**Supplementary Figures 3A, B** and Scharbert et al., 2004; Hümmer and Schreier, 2008; Nakamura et al., 2013). The absorbance of DMACA was strongest with procyanidin B2 and weakest with procyanidin A2 and poplar PAs (**Figures 1A, B**; **Supplementary Figure 3C**). The characteristic absorbance peak at 640 nm was in accordance with literature values (Wallace and Giusti, 2010), and showed slight shifts by a few nanometers for catechin, procyanidin A2 and poplar PAs towards higher wavelengths as compared to procyanidin B2 and epigallocatechin (vertical dashed lines in **Figure 1B**). Such variations had little impact on further analyses of the samples, as confirmed by regression analysis of all PA standards at varying concentration against their absorbance at 640 nm (*R* ^2^ value > 0.99, **Figure 1C**). Immediately after measuring the absorbance, fluorescence excitation and emission spectra of the same samples were recorded using a fluorimeter. Our data revealed that DMACA in the presence of all tested PAs yielded characteristic excitation maxima (*λ*_ex_max_) in the range 620 nm to 660 nm and emission maxima (*λ*_em_max_) in the range 680 nm to 700 nm, depending on the compound (**Figure 1D**). The Stokes shift (Δ*λ*) varied between 40 to 60 nm depending on the compound (**Supplementary Table 2**). With the employed equipment, the PA-specific DMACA spectra did not show any characteristic features that could be used to distinguish the respective PAs. Therefore, we measured the fluorescence intensity of PA standards of various concentrations at a constant excitation and emission wavelength (*λ*_ex_ = 650 nm, *λ*_em_ = 690 nm) and subjected the data to regression analysis. An *R* ^2^ value > 0.95 was obtained for all samples (**Figure 1E**), indicating that DMACA fluorescence was linearly proportional to the concentration of the respective PA. Since DMACA is also used as a chromophore to detect the presence of indole in samples (Miller and Wright, 1982) and indole derivatives, such as auxin (3-indoleacetic acid (IAA)), are present in plants, we measured the absorbance and fluorescence of three indole derivatives (indole, IAA and 1-naphtaleneacetic acid, NAA) (**Supplementary Figure 4**). Although indole compounds are typically present in plants at micromolar concentrations, we used a much higher concentration (1 mM) for our study. Only pure indole (not IAA or NAA) exhibited any absorbance at 640 nm and only with low intensity. Moreover, DMACA showed no indole-specific excitation and emission spectra (excitation and emission values were zero). Therefore, we concluded that there was no risk that plant indole compounds interfered with the much stronger and PA-specific DMACA fluorescence.

### Cell wall compounds do not interfere with PA-specific DMACA fluorescence but chlorophyll does

**Figure 2:**
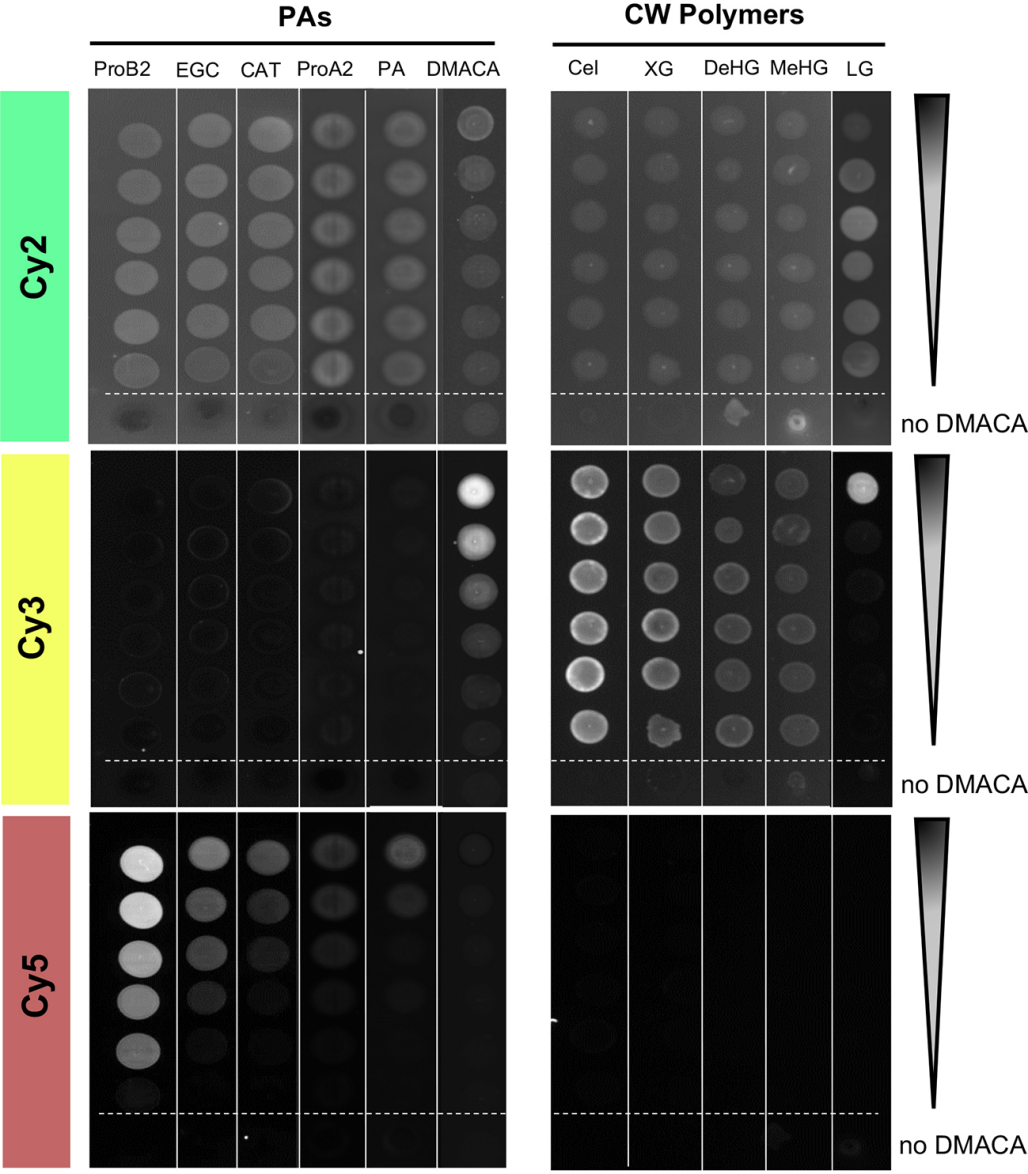
Fluorescent spot test (FST) of DMACA fluorescence (excitation *λ*_ex_, emission = *λ*_em_) with different PA species and cell wall polysaccharides. Fluorimeter settings for three channels: Cy2: *λ*_ex_= 492 nm, *λ*_em_= 510 nm; Cy3: *λ*_ex_= 550 nm, *λ*_em_= 570 nm; Cy5: *λ*_ex_= 650 nm, *λ*_em_= 670 nm. Dilutions started at 1 mg/mL for PAs and lignin and 10 mg/L for polysaccharides. At each dilution step, the concentration was halved. Arrows indicate the direction of the concentration gradient. The bottom spot (below the dashed line) in each series was recorded for only solvent (acidic alcohol, missing DMACA) with the respective substrate. ProA2, procyanidin A2; ProB2, procyanidin B2; EGC, (−)-epi-gallocatechin; CAT, (+)-catechin; PAs, proanthocyanidins from roots of *Populus tremula*; Cel, cellulose; XG, xyloglucan; DeHG, demethylesterified homogalacturonan; MeHG, methylesterified homogalacturonan and LG, lignin.

A key advantage of PA-specific fluorescence is it enables cell-wall-bound PAs to be detected in plant cells by high resolution microscopy techniques. Therefore, we examined whether DMACA exhibited any fluorescence in the presence of cell wall polymers that could potentially interfere with the PA-specific fluorescence. To overcome the limited solubility of polysaccharides in DMACA reagents, we adapted the fluorescence spot test (FST) method Thielemans et al., 2018 instead of using a liquid assay. For detection of FST results we used a western blot imaging system with three excitation and emission channels: Cy2 (*λ*_ex_/*λ*_em_= 492 nm/510 nm), Cy3 (*λ*_ex_/*λ*_em_= 550 nm/570 nm) and Cy5 (*λ*_ex_/*λ*_em_= 650 nm/670 nm). We examined the fluorescence of DMACA with cellulose, xyloglucan, homogalacturonan in two different methylesterification states and lignin (all diluted in water) Somerville et al., 2004 and included the same commercial PA standards and poplar root PAs (in DMSO) as controls. Unbound DMACA without added substrate (column 6, **Figure 2**) showed strong concentration-dependent fluorescence in the Cy3 channel, very weak to undetectable fluorescence in Cy5 and mostly non-specific background in the Cy2. Since the solvent alone (ethanol-HCl without DMACA in Cy2) had similar fluorescence as most DMACA concentrations in Cy2, most of the fluorescence in this column in Cy2 may be caused by the solvent, which was different than for all other tested compounds (**Figure 2**). There was no concentration-dependent fluorescence of the tested polysaccharides or lignin with DMACA in any of the three channels. Fluorescence observed in the Cy2 and Cy3 channels with polysaccharides or lignin was due to unbound DMACA and substrate autofluorescence. However, only in the presence of PAs, did the DMACA fluorescence shift from the Cy3 range to the expected Cy5 range, and the fluorescence intensity was PA concentration dependent. At similar concentration levels, the fluorescence intensity varied between the substrates, with the highest fluorescence observed in the presence of procyanidin B2 and the lowest in the presence of procyanidin A2 and isolated PAs. This was in line with our previous observations from spectral imaging using the fluorimeter assay. Thus, the FST assay provided a rapid, semi-quantitative visual assessment showing that PA-specific DMACA fluorescence did not interfere with cell wall polymers, polysaccharides and lignin in the relevant Cy5 range.

**Figure 3:**
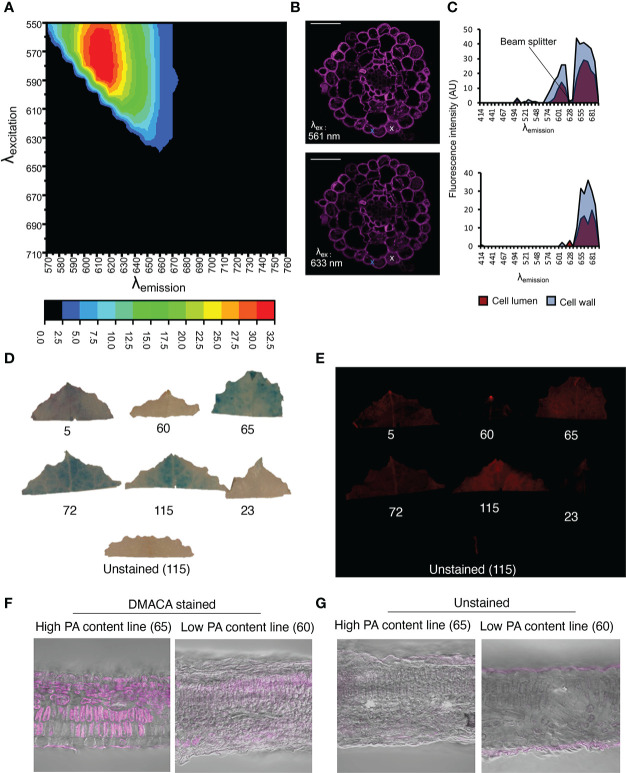
PA detection by DMACA detection in Populus root and leaf material. The relative fluorescence intensity of DMACA-stained poplar roots depends on the selected excitation and emission wavelengths: **(A)** Contour plot showing the relative fluorescence intensity (colored scale) for various combinations of excitation and emission wavelength (*λ*) acquired through a lambda-lambda scan with a tunable white light laser. **(B)** DMACA-stained poplar root images from two lambda scans upon excitation with two commonly used confocal laser lines 561 nm and 633 nm (within the excitation area shown in the contour plot). Scale bars = 50 µm. **(C)** Comparison of tissue spectral representations of cell lumen and cell wall areas excited with 561 nm (upper graph) and 633 nm (lower graph) laser lines. The spectra were identified with linear unmixing tools in Zeiss Zen software by manually using the cross tool to mark the cell lumen and cell wall areas. The spectra of vacuolar PAs and cell-wall-bound PAs showed comparable emission ranges in the red region of the spectrum, as shown in **Figure 1**. **(D–G)** Detection of PAs with DMACA in *Populus tremula* genotypes from the Swedish Aspen Collection (SwASP) with different leaf PA levels (Bandau et al., 2015). The ID number corresponds to the SwAsp ID. **(D)** Light images showing PA levels as blue precipitate. **(E)** DMACA-stained leaves upon excitation and imaging with a fluorescent imaging system (Azure 600c, Azure Biosystems) using the red channel (λ_ex_/λ_em_= 650 nm/670 nm). Leaf section of *P. tremula* lines with high and low PA content stained with DMACA **(F)** and imaged under the confocal microscope, overlay with brightfield images. Unstained sections showing background fluorescence **(G)**. Scalebar in **(F–G)** 50µm.

Although *in vitro* investigations with the fluorimeter and FST method showed that the PA-specific DMACA excitation and emission spectra lay within the red portion of the light spectrum, aberrations in these spectra can occur in DMACA-stained plant tissue at *in situ* level. Therefore, we performed a simultaneous excitation and emission scan of DMACA-stained poplar root (containing PAs) cross-sections using a tunable white-light laser and confocal microscope (**Figure 3**). The detected excitation range was 510 to 640 nm, which was broader than the excitation range of DMACA with isolated poplar PAs under *in vitro* conditions. In contrast, the emission range was between 670 and 692 nm, similar to that recorded for the *in vitro* conditions tested above. A slight change in the excitation and emission ranges between *in vitro* and *in situ* conditions was to be expected due to the more complex environment of PAs inside plant tissues, which can influence the fluorescence of compounds. Our results suggest that DMACA fluorescence should ideally be used with 561 nm or 633 nm laser lines, which are commonly applied in confocal microscopy (**Figure 3**). Conveniently, the relevant excitation and emission ranges do not interfere with autofluorescence spectra of other plant polyphenolic compounds, which typically lie below these ranges (Talamond et al., 2015, Donaldson, 2020). However, since the emission range of PA-specific DMACA fluorescence overlaps with chlorophyll autofluorescence (Talamond et al., 2015), caution must be exercised when analyzing chlorophyll containing tissue as our experiments with leaves from *Populus tremula* genotypes from the Swedish Aspen Collection (SwASP) shows. These leaves have different levels of PAs (Bandau et al., 2015). The leaves were stained with DMACA reagent following the procedure described in materials and methods. We compared brightfield images to fluorescence images (**Figure 3**), since DMACA is known to form a blue/purple precipitate when bound to PAs Li et al., 1996. PA levels differed among the genotypes as indicated by the presence of blue colour from DMACA staining in brightfield images. Upon excitation and imaging with a fluorescent imaging system (**Figure 3E**) using the red channel (λ_ex_/λ_em_= 650 nm/670 nm), fluorescence was observed mainly in high tannin areas in the leaves (corresponding to areas of blue color in **Figure 3D**). In a few areas, red fluorescence was observed, which does not correspond to blue color in **Figure 3D**, e.g. in leaf 115, in the vein of leaf 5 and 72 and at the leaf tip (potentially a hydatode) of leaf 5 and 60. This may be due to the presence of uncleared chlorophyll and the presence of other autofluorescent compounds in the vascular systems, since we also see some faint fluorescence in the unstained leaf’s vasculature. Similar results, with background fluorescence probably from uncleared chlorophyll and/or cuticular waxes, were observed in leaf sections (**Figures 3F, G**). This highlights the importance of using appropriate non-stained controls to distinguish DMACA fluorescence from autofluorescence of other compounds especially in materials that contain chlorophyll, waxes or other compounds with red autofluorescence in the red spectral region.

### PA-specific DMACA spectral properties are suitable for fluorescence microscopy

We investigated the suitability of the DMACA fluorophore for use in fluorescence microscopy, specifically for the *in situ* localization of PAs in root tissue. Regions of blue DMACA-PA precipitate observed with a light microscope overlapped with fluorescent regions observed with an epi-fluorescence microscope (**Supplementary Figure 5**), indicating that the fluorescence was representative of PAs in root tissue. Especially in the epidermis cells we observed intracellular precipitates overlapping with fluorescence. The absence of such intracellular contents in certain cells may result from the fact that cells are cut open during vibratome-sectioning and cell-contents are lost. Interestingly, cell-wall-bound fluorescence was more prominent in fluorescence images than the blue/purple coloration in brightfield images, suggesting that DMACA fluorescence is a sensitive way of visualizing cell-wall-bound PAs.

**Figure 4:**
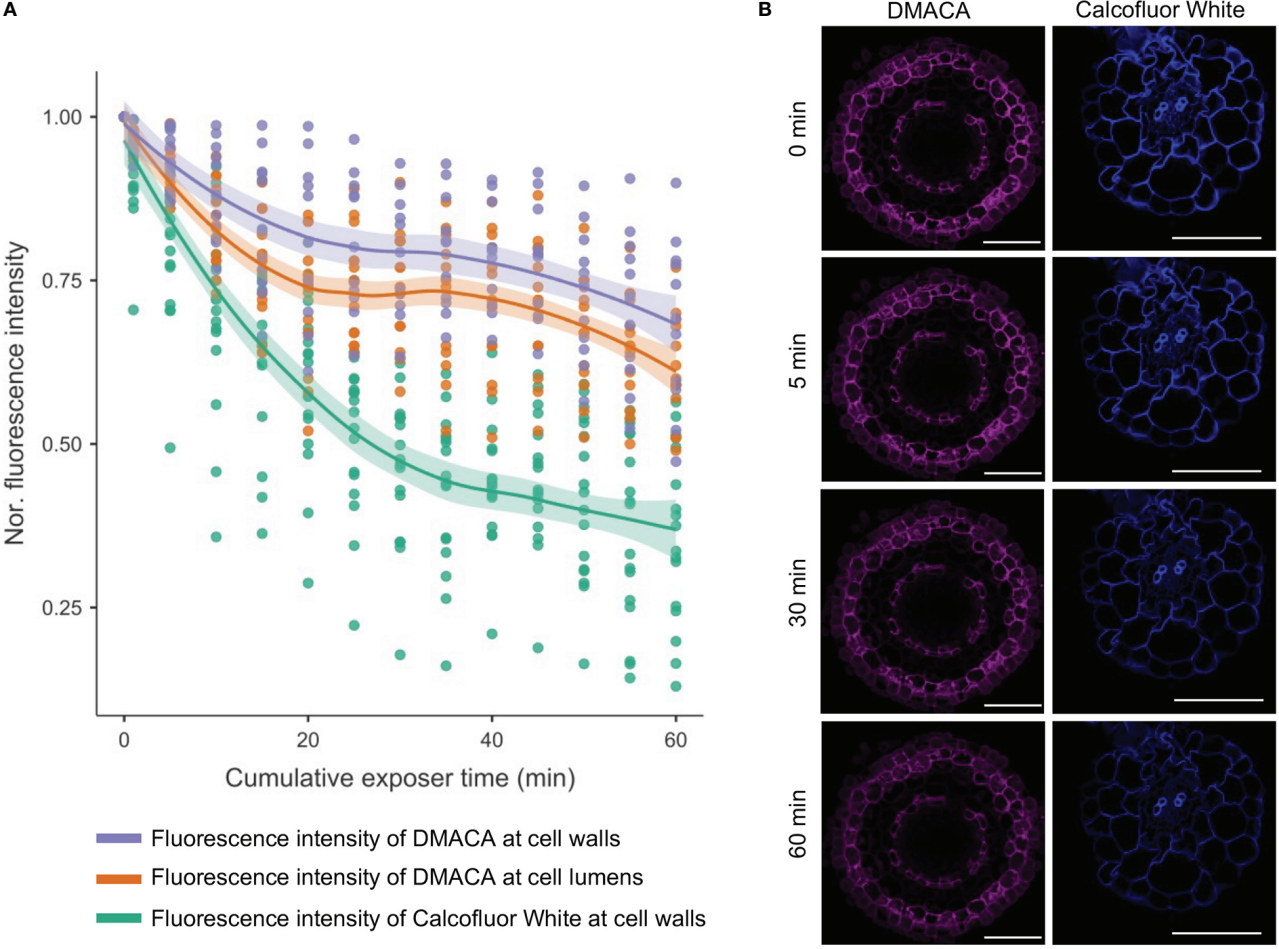
Photobleaching curves for Calcofluor White (excited with a 405 nm laser) and DMACA (excited with a 633 nm laser) stained poplar roots. **(A)** Line graph showing the average signal intensity of various areas of plant cells normalized to the signal at timepoint zero. The shaded areas represent 95% confidence intervals. **(B)** Micrographs of poplar root cross-sections at 0, 5, 30 and 60 minutes after scanning, showing photobleaching of samples stained with DMACA and Calcofluor White. The laser gain was slightly different for the two lasers but was maintained constant over time. All other scan parameters were the same for the two laser lines. Scale bars = 50 µm.

To determine the analytical stability of DMACA fluorescence in root tissues, we performed a photobleaching experiment including Calcofluor White for comparison, which is a commonly used cell wall fluorophore. Bleaching of the DMACA fluorescence intensity was much slower than that of Calcofluor White at their corresponding excitation wavelengths (405 nm for Calcofluor White and 633 nm for DMACA) (**Figure 4**). After 5 minutes of continuous exposure, the DMACA fluorescence intensity of the cell wall and lumen decreased by less than 10% of the original intensity, whereas the Calcofluor White fluorescence intensity decreased by about 20%. After 30 minutes and 60 minutes of continuous scanning, the DMACA fluorescence intensity had decreased by approximately 25% and 35%, respectively, whereas Calcofluor White fluorescence decreased by approximately 60% and 75%, respectively. This indicated that the PA-specific DMACA fluorescence signal was highly stable.

**Figure 5:**
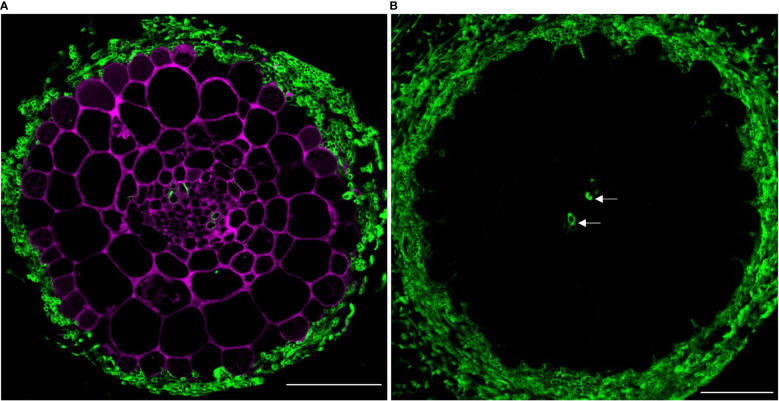
**(A)** *In situ* co-localization of PAs and chitin in the ectomycorrhizal fungus *Laccaria bicolor* in a lateral root cross-section (taken 500 µm from the root tip) of *Populus tremula* x *Populus tremuloides*. PAs in the root (DMACA staining; magenta) surrounded by a mantle of the symbiont (wheat germ agglutinin (WGA) staining; green) circa seven days into the colonization phase. **(B)** Similarly aged root stained with WGA only and without DMACA (negative control). To our observation, other than the fungal cell wall, WGA also binds to the xylem wall (indicated with arrow). Scale bar = 50 µm. The experiment was performed on 3-4 roots originating from separate plants yielding similar results in replicates.

Next, we assessed the compatibility of PA-specific DMACA fluorescence with other dyes with green and blue fluorescence. In the first example, we used poplar roots colonized by an ectomycorrhizal fungus and stained them with DMACA, which binds to plant cell walls as reported above, and Alexa Fluor 488 conjugated wheat germ agglutinin (WGA), which binds to chitin in fungal walls (Peters and Latka, 1986) (**Figure 5A**). Negative controls without DMACA did not show any signal in the red area of the spectrum (**Figure 5B**), showing that the dye is more suitable for use in root as compared to leaf tissues (**Figure 3**). The results confirmed the compatibility of the two dyes and specific localization to plant (DMACA) and fungal (WGA) cell walls.

### High-resolution imaging of DMACA-stained poplar roots reveals cell-wall-bound localization features of PAs

**Figure 6:**
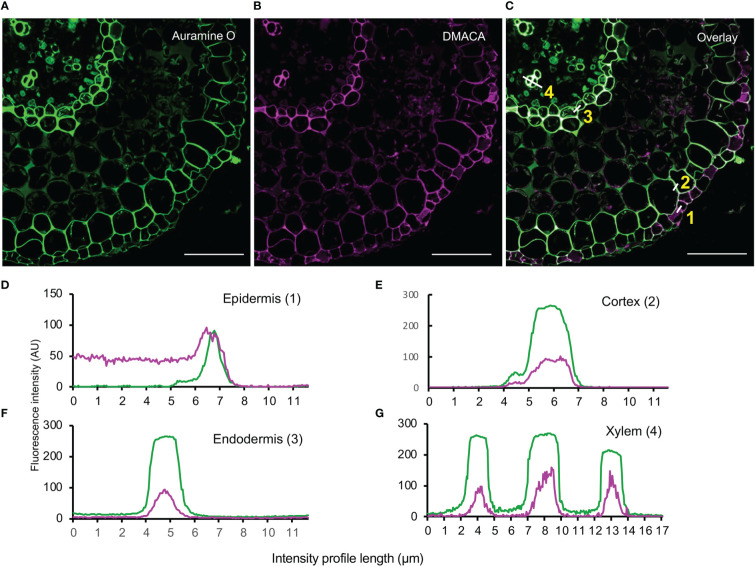
Double staining with **(A)** Auramine O (green) and **(B)** DMACA (magenta) on cross-sections (taken at 500 µm from the root tip) of 28-day-old *Populus tremula* x *Populus tremuloides* roots. **(C)** Overlay showing *in situ* localization of lignified (xylem) and suberized areas (endodermis and cortex) together with PAs in lateral root cross-sections. Scale bars = 50 µm. **(D–G)** Fluorescence intensity profiles across cell walls in four cell layers (white lines in **(C)**) showing overlapping fluorescence of Auramine O (green) and DMACA (magenta) in epidermis, cortex, endodermis and xylem cell walls. The experiment was performed on 3-4 roots originating from separate plants yielding similar results in replicates.

To investigate co-localization of cell wall polymers with PAs, we counterstained DMACA-stained hybrid aspen lateral roots with Auramine O, a green fluorescent dye that binds to lignin, suberin and cutin (Nishikawa et al., 2005; Pesquet et al., 2005; Ursache et al., 2018). Intense fluorescence signals were observed for both DMACA and Auramine O in the longitudinal wall of the epidermis, first cortex layer, endodermis and xylem vessel in cross-sections taken 500 µm from the root tip in 28-day-old lateral roots (**Figure 6**). Fluorescence intensity profiles showed significant overlap of the DMACA and Auramine O fluorescence in these areas. However, in section close to the root tip (300 µm) through newly emerged (three day old) lateral roots, we observed an absence of DMACA fluorescence in the Casparian strip, which was well stained with Auramine O (**Supplementary Figure 6**). Since Casparian strips develop by deposition of suberin at an early developmental stage of the endodermis (Kirkham, 2014; Andersen et al., 2015) our data indicated that PAs are incorporated into the endodermis at a later stage. Lignification is a common feature of the xylem wall (Lourenço et al., 2016), which probably explains the intense Auramine O staining in such cells (**Figure 6**). Since DMACA does not exhibit lignin-specific fluorescence (**Figure 2**), the presence of DMACA fluorescence in the xylem wall suggests that PAs were incorporated in the xylem wall as well. Therefore, use of DMACA as a fluorescent stain may facilitate investigations of possible connections of PAs with lignin (Dixon and Sarnala, 2020) and other polymers in cell walls, e.g., suberin.

**Figure 7:**
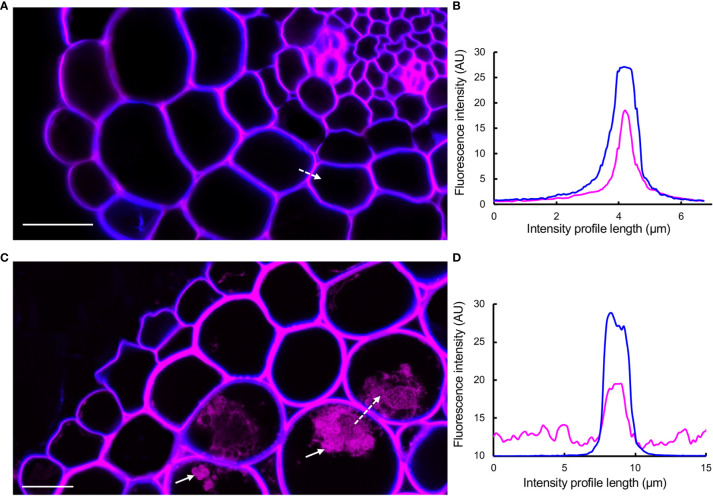
DMACA (magenta)/Calcofluor White (blue) double staining detected by Airyscan microscopy revealing the location of PAs in cell walls and intracellular areas in lateral root cross-sections (taken 500 µm from root tip) of *Populus tremula* x *Populus tremuloides*. **(A)** Young root at 7 days after emergence (dae). **(B)** Graph representing intensity profiles of DMACA (magenta-colored lines) and Calcofluor White (blue-colored lines) measured along the dashed white arrow in **(A)**. **(C)** Old lateral root at 28 dae. **(D)** Graph representing intensity profiles of DMACA and Calcofluor White measured along the dashed white arrow in **(C)**. The solid white arrows in **(C)** show the presence of PAs intracellularly. Scale bars = 20 µm. The experiment was performed on 3-4 roots originating from separate plants per time point yielding similar results as shown above.

Studies using macerated cell walls have shown that PAs have a high binding affinity to pectic polysaccharides, potentially through non-covalent bonds (Riou et al., 2002; Watrelot et al., 2013; Renard et al., 2017; Zhu, 2018; Liu et al., 2020; Watrelot and Norton, 2020; Liu et al., 2021). Using DMACA and Calcofluor White as a cell wall counterstain (Wood, 1980; Mori and Bellani, 1996) in combination with Airyscan mode microscopy that increases signal-to-noise ratio and resolution (Huff, 2015) on cross-sections of 7- and 28-day-old (after emergence, dae) lateral roots, we investigated whether PAs specifically localize to the middle lamella between adjacent cell walls. This area is rich in pectin (Daher and Braybrook, 2015), and potential localization of PAs in the middle lamella may strengthen the hypothesis that PAs and pectins may interact *in muro*. The micrographs (**Figure 7**) revealed that in young roots (7 dae), the DMACA fluorescence intensity was highest near the middle lamella between two adjacent cells and the fluorescence intensity decreased towards the cell lumen. In older roots (28 dae), the DMACA fluorescence was more evenly distributed over the cell wall, overlapping largely with Calcofluor White fluorescence. These observations suggest that in young lateral roots, PAs localize more specifically to areas of high pectin content in the middle lamella (Daher and Braybrook, 2015). However, the localization may be dynamic, and during aging, PA deposition may increase (as concluded from stronger signals in old roots) and spread to the primary wall.

## Discussion

We characterized the fluorogenic properties of DMACA toward PAs, which are commonly found in plant tissues. DMACA is a widely used chromophore for PA detection owing to its high sensitivity and reliability (Hümmer and Schreier, 2008) but has hitherto not been characterized as a fluorescent dye for diverse fluorescence-based microscopy techniques. However, cinnamic acid derivatives, including DMACA, exhibit fluorogenic properties that have been exploited in various fields of material sciences (Dryjanski et al., 1999; Singh and Mitra, 2008; Fritz et al., 2015). It has been proposed that, under acidic conditions such as used in our study, DMACA preferentially reacts with the terminal units of proanthocyanidin, favoring the C8 position of the A-ring (Wallace and Giusti, 2010). The literature provides some information about the fluorescence mechanisms of DMACA alone, in the absence of PAs (Bangal et al., 2001). DMACA and its derivatives have also been used to create fluorescence probes by reacting with various compounds, which can form a double twistable ethylene structure with intramolecular charge transfer capabilities and hence, emit fluorescence in the red to infrared spectra upon photoexcitation (Chen et al., 2021; Gao et al., 2022; Sheshashena Reddy and Ram Reddy, 2013). However, further studies are needed to understand the chemistry and physics of the DMACA fluorescence shift with its substrates and this was beyond the scope of our study oriented to the biological use of DMACA in plant tissues. Our data demonstrated that DMACA exhibits PA-specific fluorescence that can be used to reveal important localization attributes of both intracellular and cell-wall-bound PAs through combination with other fluorogenic dyes and microscopy techniques.

We revealed that PA-specific DMACA fluorescence lies in the red light region of the spectrum, which we confirmed by *in vitro* determination using commercial and isolated PAs commonly present in plants and by *in situ* determination of hybrid aspen roots known to be rich in PAs (Dixon et al., 2005). A slight difference in the emission and excitation ranges was observed between the *in vitro* and *in situ* studies, which was expected since several physicochemical parameters, including pH and electrostatic interactions with other molecules, can affect fluorescence spectra (Paës, 2014), in addition to instrumental differences. In addition, differences in the polymer length of PAs may affect DMACA absorbance (Wang et al., 2016) and fluorescence. Since the DMACA fluorescence spectrum appears to be both solvent- and substrate-dependent because of its intramolecular charge transfer properties (Bangal et al., 2001; Singh and Mitra, 2008), we recommend that, if different solvents are used, the DMACA fluorescence spectrum should be verified prior to conducting biological experiments. Since one of our goals was to localize cell-wall-bound PAs *in situ*, we investigated whether DMACA exhibits cell wall polymer-specific fluorescence that interferes with PA-specific fluorescence. A fluorophore’s fluorescence emission may be enhanced or shifted after selectively binding with cell wall polysaccharides. For example, Trypan Blue, Solophenyl Flavine 7GFE and Pontamine Fast Scarlet 4B change emission properties after binding selectively to glycan, cellulose and xyloglucan, respectively (Anderson et al., 2010; Liesche et al., 2015). Using a fluorescence spot test (FST), we confirmed that DMACA yielded a red emission spectrum only in the presence of PAs and not in the presence of cell wall polysaccharides or lignin (**Figure 2**). Therefore, the PA-specific DMACA fluorescence should be similar for both wall-bound PAs and vacuolar PAs in plant tissue. This finding was consistent with our subsequent *in situ* study, where we found that the fluorescence spectral pattern was similar in intracellular content and cell wall area (**Figure 3**). To draw exact conclusion on the nature of intracellular structures, the fixation and staining protocol would need to be modified to preserve organelles. By measuring several spectral properties, we showed that PA-specific DMACA fluorescence is suitable for confocal microscopy. PA-specific DMACA fluorescence spectra have a Stokes shift of about 40 nm to 60 nm, which is large enough to separate excitation light from emission light to obtain a high signal-to-noise ratio (Gao et al., 2017; Ren et al., 2018). PA-specific DMACA fluorescence was also found to be stable even under long-term observation. To the best of our knowledge, the DMACA fluorescence signal stability has not been assessed before in any study. Therefore, we could not compare the DMACA signal stability in the presence of other compounds. DMACA as a fluorophore is also convenient as two commonly available confocal laser lines, 561 nm and 633 nm, are suitable for excitation of DMACA-stained plant tissues, such as hybrid aspen roots. However, excitation with a 561 nm laser yielded broader emission in the red spectrum compared to excitation with a 633 nm laser (**Figure 3**).

We demonstrated several potential uses of PA-specific DMACA fluorescence in combination with other fluorescent dyes. Using Alexa Fluor 488 conjugated wheat germ agglutinin (WGA), which binds fungal chitin, we demonstrated localization of PAs in plant roots colonized by fungal mycelia during hybrid aspen-ectomycorrhizal fungal interactions (**Figure 5**). We are currently investigating the dynamics of host root PAs during ectomycorrhizal symbiosis (Ferdous, Chowdhury et al. unpublished) using DMACA in combination with confocal microscopy. Such a dual staining technique could be used to investigate other plant-microbe interactions to understand PA functions, since PAs are known to have antimicrobial properties (Scalbert, 1991). Furthermore, we used confocal microscopy in Airyscan mode to examine double-stained tissue to improve the spatial resolution and signal-to-noise ratio without increasing the laser excitation power and image acquisition time (Korobchevskaya et al., 2017). This supported our findings from *in vitro* studies that PAs can bind cell wall compounds and such binding patterns may change over time during tissue development and maturation (**Figure 7**). Similarly, we showed that wall-bound PAs colocalize with wall-bound phenolics, such as lignin and suberin, as revealed by Auramine O staining in root tissue (**Figure 6**). Our current protocol for PA-specific DMACA fluorescence imaging is best applicable for tissues devoid of chlorophyll, which has an overlapping emission range with the PA-specific DMACA fluorescence (Donaldson, 2020) and could confound the interpretation of the red fluorescence. Therefore, when staining above-ground tissues, special care needs to be taken. One solution is to extend the ethanol washes that are part of the staining protocol to clear tissues from chlorophyll. Another solution can be the analysis of fluorescence lifetime of the fluorescence, which will differ between PA-specific DMACA and chlorophyll-dependent autofluorescence. This requires however a microscope equipped with tools for fluorescence lifetime analysis. In any case, it is strongly advised to always compare the PA-specific DMACA signal to non-stained control samples treated with the reagents without DMACA.

Our study shows for the first time that DMACA fluorescence can be used to visualize PAs in plant cell walls and its colocalization with cell wall polymers, providing a novel tool to study PA interaction with various cell wall polymers and PA dynamics on a sub-cellular level. While the current investigation is limited to a co-localization study, it opens the possibility of further study into how these cell-wall-bound PAs interact with other polymers, including pectin, lignin and suberin. The methods can be modified to serve specific purposes. For example, fluorescence-lifetime imaging microscopy (FLIM) techniques could be used to determine whether there is a distinguishable DMACA spectra for different PA standards. PA-specific DMACA fluorescence may be compatible with other important fluorescent plant cell dyes that emit light in the blue and green spectrum. Furthermore, attempts could be made to incorporate DMACA staining in immunolocalization techniques based on the high sensitivity of antibodies toward their substrates, but will require adaptation of sample fixation so to include formaldehydes for epitope conservation. Such combinations may not only improve the spatial visualization of specific PA and wall polymer connections but also provide a vital tool for deconstructing complex cell wall polymers in the future. Taken together, our results demonstrate that fluorescence microscopy techniques provide new and exciting avenues for studying PAs *in planta*, including PA polymerization and degradation. This may facilitate cell biological studies and analyses of genotypic differences in PA biosynthesis and storage, as well as research into the effects of ontogeny and environmental impacts of interest in chemical ecology and plant physiology, using *Salicaceae* trees as model systems (Bailey et al., 2005; Barbehenn and Peter Constabel, 2011; Bandau et al., 2015; Dettlaff et al., 2018; Ullah et al., 2019; Bandau et al., 2021; Cole et al., 2021).

## Data availability statement

The original contributions presented in the study are included in the article/**Supplementary Material**. Further inquiries can be directed to the corresponding authors.

## Author contributions

JC, BA and JL-F designed the study. JC conducted the experiments and analyzed the data. JL-F performed spectral analysis and supervised the study. JF and JL isolated proanthocyanidins from poplar roots. JC and JL-F wrote the manuscript with contributions from all authors.
