# Supplementary material for: Fluorogenic properties of 4-dimethylaminocinnamaldehyde (DMACA) enable high resolution imaging of cell-wall-bound proanthocyanidins in plant root tissues
Source: Front Plant Sci. 2023 Jan 16;13:1060804. doi: 10.3389/fpls.2022.1060804 (PMC9884812; doi:10.3389/fpls.2022.1060804)
Supplement: Supplementary file 1 [file DataSheet_1.pdf]

Supplementary Material

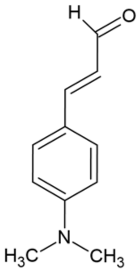

4-(Dimethylamino)cinnamaldehyde

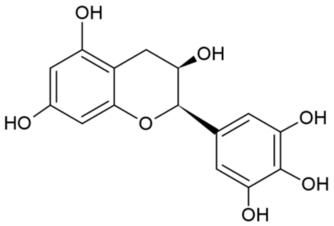

(−)-Epigallocatechin

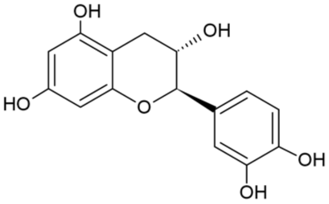

(+)-Catechin

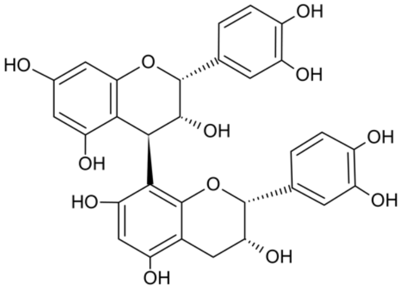

Procyanidin B2

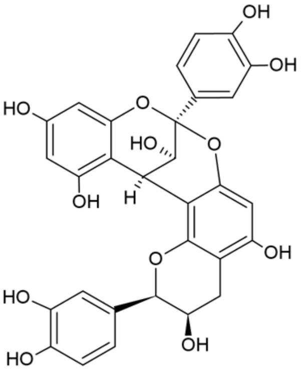

Procyanidin A2

Supplementary Figure 1: Chemical structures of DMACA and PAs used in this study. The structures were drawn using ChemSketch software (ACD Labs) with the respective SMILES ID provided by product vendors (Sigma-Aldrich, Sweden AB, Stockholm). The catalogue numbers of the compounds are given in the materials and methods section.

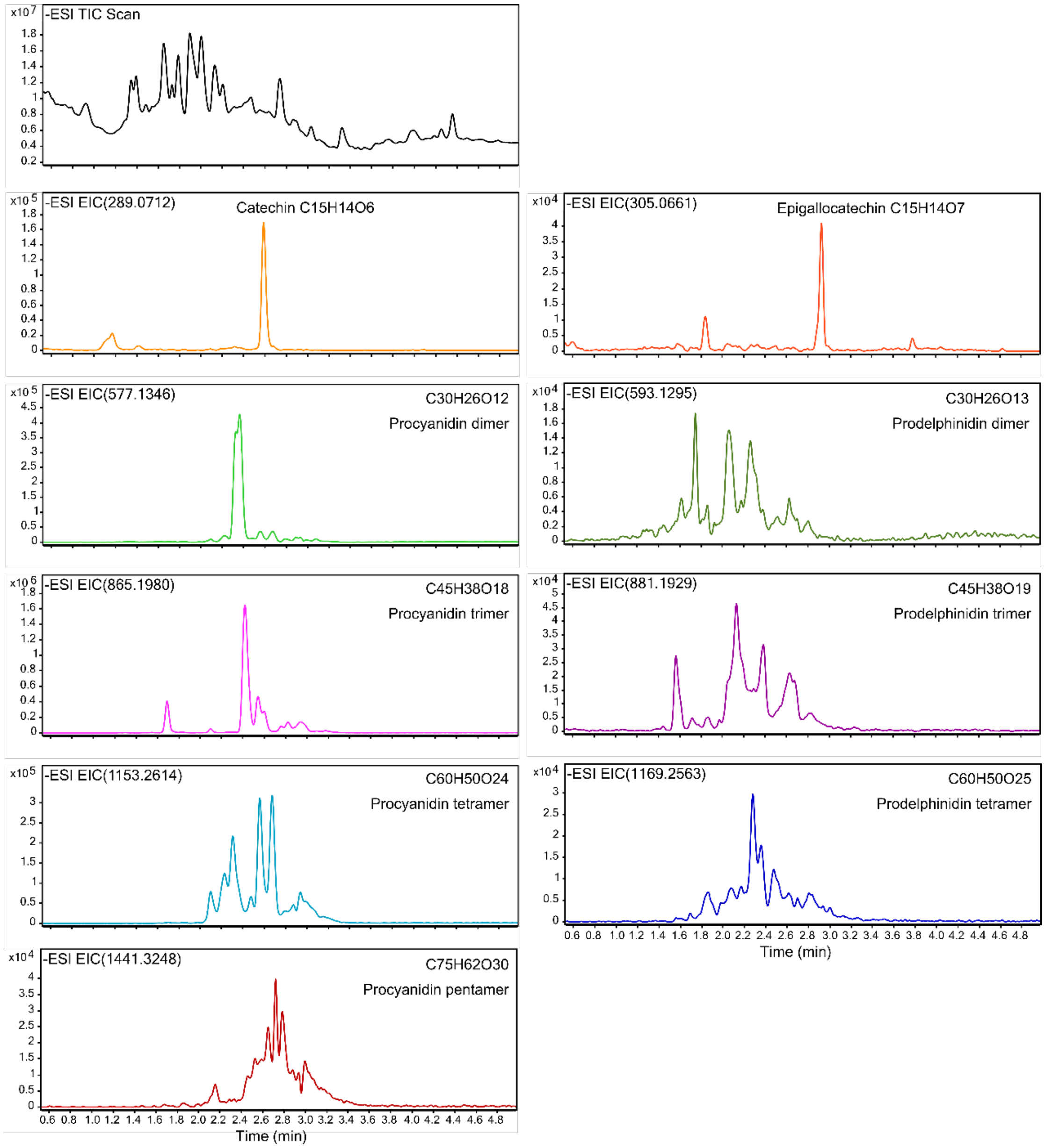

Supplementary Figure 2. Total ion chromatogram (TIC) and selected extracted ion chromatograms (EIC) of proanthocyanidin (PA) fraction isolated from hybrid aspen roots. The extracted mass, the corresponding molecular formula and the tentatively identified PAs are shown beside the traces. Each peak corresponds to an isomer of the molecular formula. Data are from negative ionization mode. See Supplementary table 1 for a list of detected PAs.

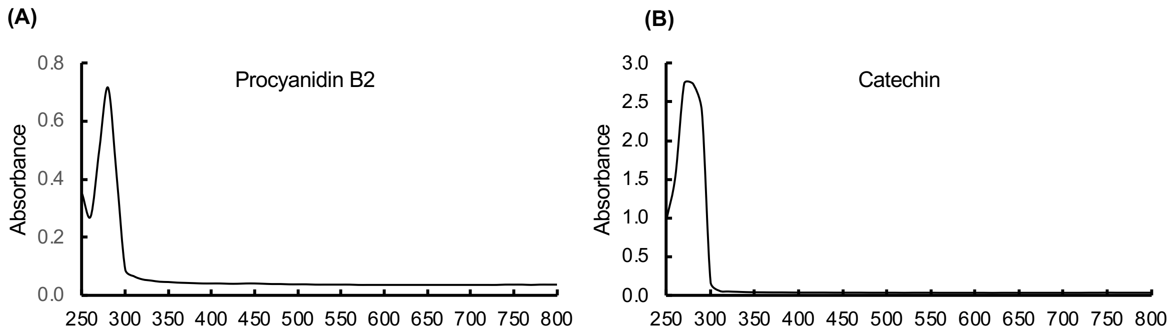

Supplementary Figure 3. Absorbance of Procyanidin B2 and Catechin in the absence of DMACA (A, B) and scale adjusted characteristics absorbance of DMACA in the presence of PAs (C, D). Absorbance spectra of procyanidin B2 (A) and catechin (B) in EtOH-HCl in the absence of DMACA recorded between 250 and 800nm.

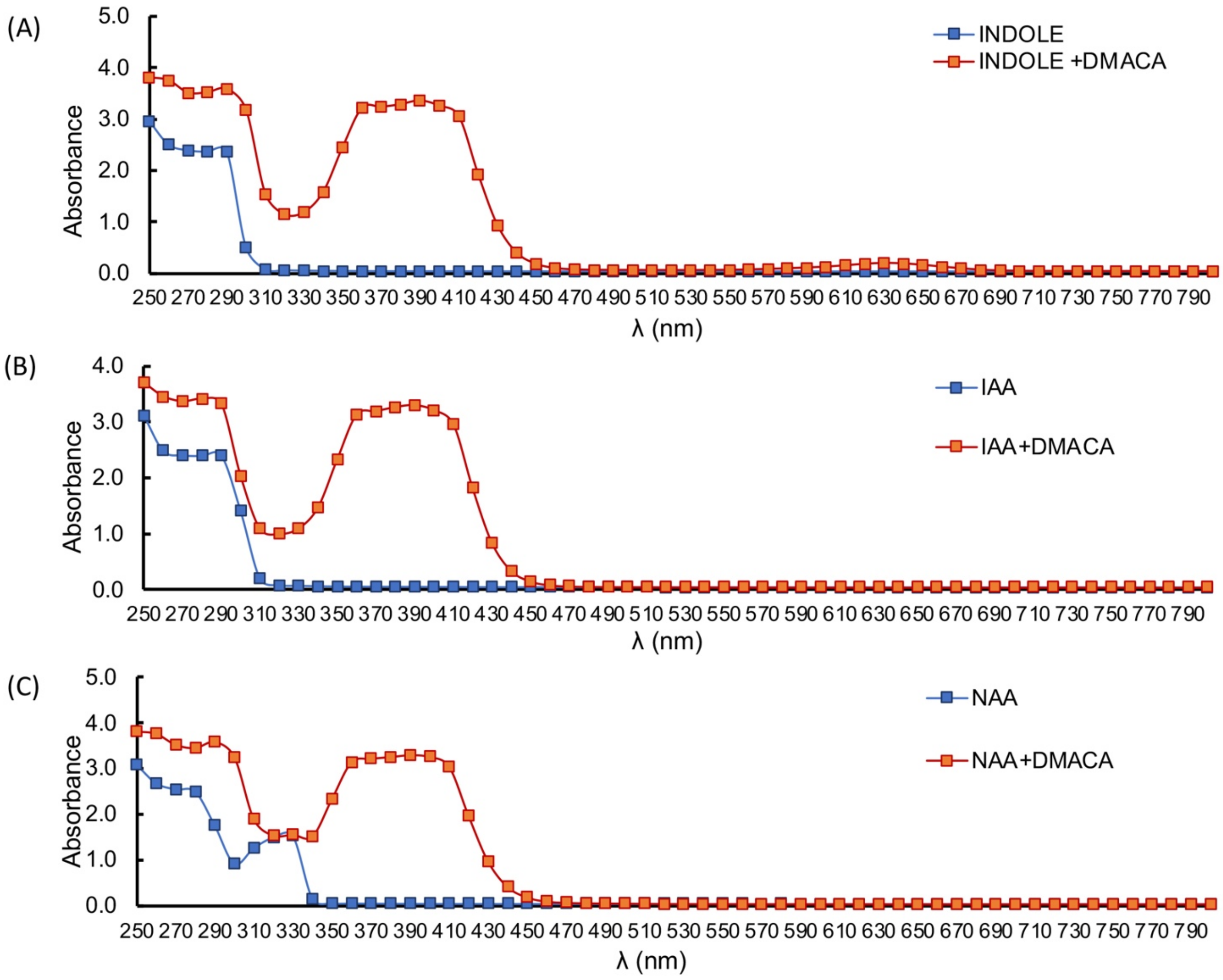

Supplementary Figure 4. Absorbance spectra of indole and plant indole compounds at high concentration (1 mM) in acidic alcohol media with or without DMACA. Although indole showed slightly increased absorbance at 640 nm (A), its naturally occurring plant derivative indole-3-acetic acid (IAA) (B) and synthetic derivative 1-naphthaleneacetic acid (NAA) (C) showed no increase of absorbance at 640 nm, suggesting that interference of indole compounds with DMACA is negligible in plants.

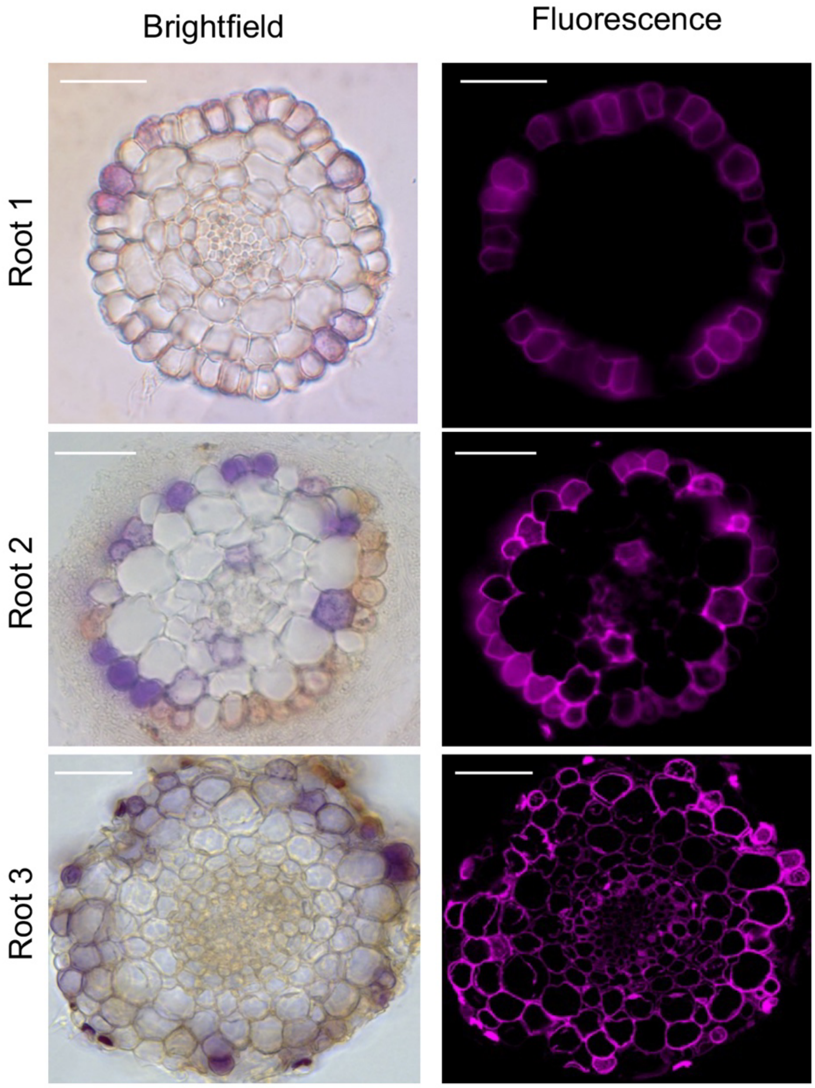

Supplementary Figure 5. Chromophore and fluorophore comparison of DMACA-stained *Populus tremula* x *Populus tremuloides* root sections. The first column shows brightfield images of root cross-sections from three different plants. Blue/purple color in the brightfield images indicates the presence of PAs in the plant cells. The second column shows fluorescence images of corresponding sections under an epi-fluorescence microscope using a Y5 filter cube (Ex. 590-650 nm, dichroic mirror 660 nm, Em. 662-738 nm).

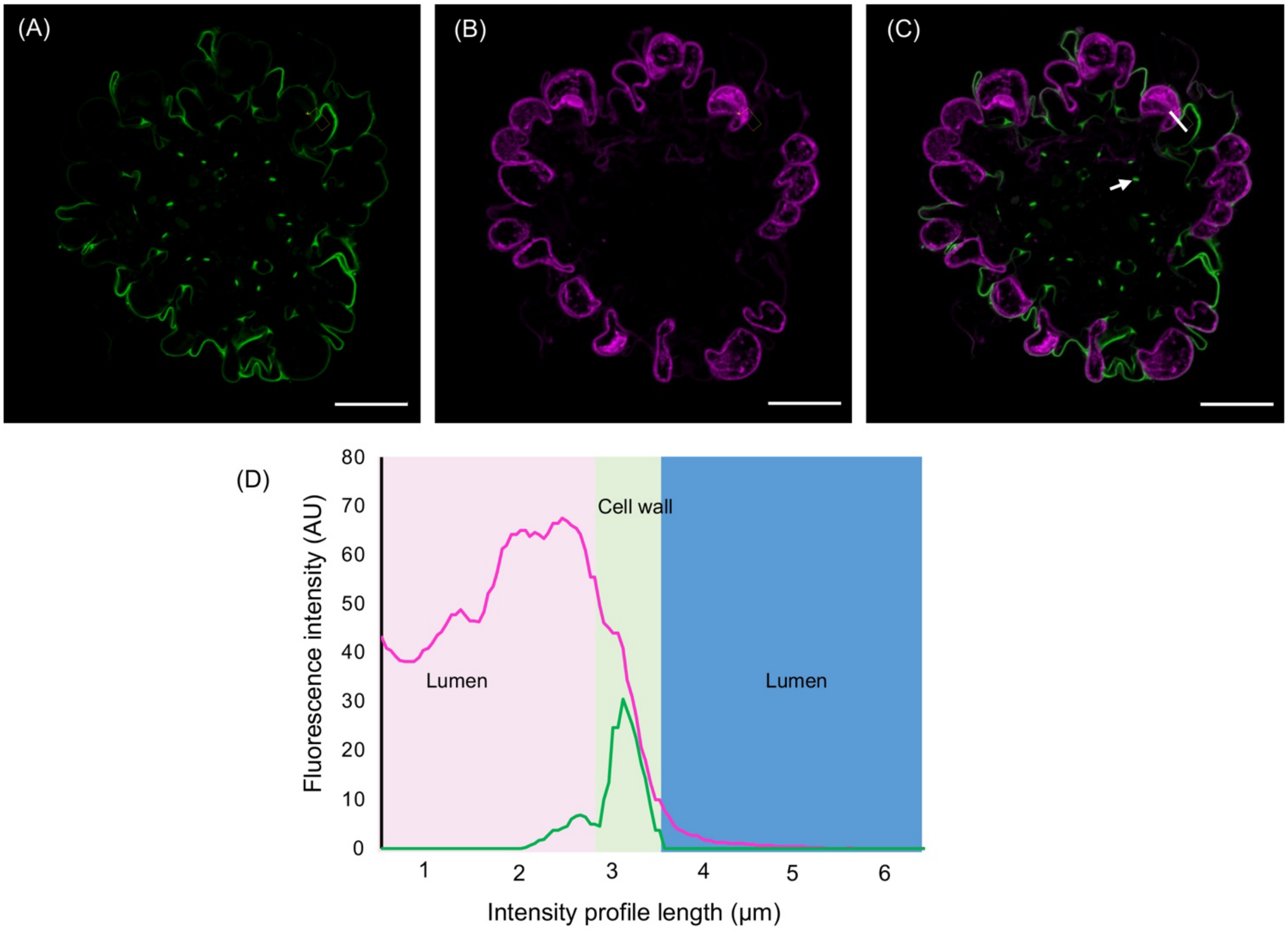

Supplementary Figure 6. Auramine O (A) – DMACA (B) double staining in root tips of a newly emerged lateral root (3 days after emergence) cross-section (taken at 300 µm from the root tip) of *Populus tremula* x *Populus tremuloides*. (C) Overlay image of Auramine O (green) and DMACA (magenta) staining. Scale bars = 200 µm. (C) Fluorescence intensity profile recorded along the white line showing the overlapping fluorescence intensity of Auramine O (green) and DMACA (magenta) in the epidermal wall. The arrow in (C) indicates the Casparian strip where no DMACA fluorescence was found. Images are one representative example of pictures taken of 3-4 roots from different plants.
